# Comparative Effect of Collaborative Care, Pain Medication, and Duloxetine in the Treatment of Major Depressive Disorder and Comorbid (Sub)Chronic Pain: Results of an Exploratory Randomized, Placebo-Controlled, Multicenter Trial (CC:PAINDIP)

**DOI:** 10.3389/fpsyt.2018.00118

**Published:** 2018-04-05

**Authors:** Eric W. de Heer, Jack Dekker, Aartjan T. F. Beekman, Harm W. J. van Marwijk, Tjalling J. Holwerda, Pierre M. Bet, Joost Roth, Lotte Timmerman, Christina M. van der Feltz-Cornelis

**Affiliations:** ^1^GGz Breburg, Clinical Centre of Excellence for Body, Mind and Health, Tilburg, Netherlands; ^2^Tranzo Department, Tilburg School of Behavioral and Social Sciences, Tilburg University, Tilburg, Netherlands; ^3^Faculty of Behavioral and Movement Sciences, VU University, Amsterdam, Netherlands; ^4^Arkin, Mental Health Institute, Amsterdam, Netherlands; ^5^Department of Psychiatry, VU University Medical Centre, Amsterdam, Netherlands; ^6^GGz inGeest, Mental Health Institute, Amsterdam, Netherlands; ^7^EMGO Institute for Health and Care Research (EMGO+), Amsterdam, Netherlands; ^8^Department of General Practice, VU University Medical Centre, Amsterdam, Netherlands; ^9^Division of Population Health, Health Services Research and Primary Care, University of Manchester, Manchester, United Kingdom; ^10^Department of Clinical Pharmacology and Pharmacy, VU University Medical Centre, Amsterdam, Netherlands

**Keywords:** collaborative care, pain, depression, duloxetine, placebo, pregabalin, algorithm

## Abstract

**Objective:**

Evidence exists for the efficacy of collaborative care (CC) for major depressive disorder (MDD), for the efficacy of the consequent use of pain medication against pain, and for the efficacy of duloxetine against both MDD and neuropathic pain. Their relative effectiveness in comorbid MDD and pain has never been established so far. This study explores the effectiveness of CC with pain medication and duloxetine, and CC with pain medication and placebo, compared with duloxetine alone, on depressive and pain symptoms. This study was prematurely terminated because of massive reorganizations and reimbursement changes in mental health care in the Netherlands during the study period and is therefore of exploratory nature.

**Methods:**

Three-armed, randomized, multicenter, placebo-controlled trial at three specialized mental health outpatient clinics with patients who screened positive for MDD. Interventions lasted 12 weeks. Pain medication was administered according to an algorithm that avoids opiate prescription as much as possible, where paracetamol, COX inhibitors, and pregabalin are offered as steps before opiates are considered. Patients who did not show up for three or more sessions were registered as non-compliant. Explorative, intention-to-treat and per protocol, multilevel regression analyses were performed. The trial is listed in the trial registration (http://www.trialregister.nl/trialreg/admin/rctview.asp?TC=1089; NTR number: NTR1089).

**Results:**

Sixty patients completed the study. Patients in all treatment groups reported significantly less depressive and pain symptoms after 12 weeks. CC with placebo condition showed the fastest decrease in depressive symptoms compared with the duloxetine alone group (*b* = −0.78; *p* = 0.01). Non-compliant patients (*n* = 31) did not improve over the 12-week period, in contrast to compliant patients (*n* = 29). Pain outcomes did not differ between the three groups.

**Conclusion:**

In MDD and pain, patient’s compliance and placebo effects are more important in attaining effect than choice of one of the treatments. Active pain management with COX inhibitors and pregabalin as alternatives to tramadol or other opiates might provide an attractive alternative to the current WHO pain ladder as it avoids opiate prescription as much as possible. The generalizability is limited due to the small sample size. Larger studies are needed.

## Introduction

Pain is common among depressive patients (1–4), with comorbidity rates amounting to two thirds (5). A depressive disorder is a risk factor for developing low-back pain (6, 7), neck pain (6), and joint pain (8). The burden of depression with comorbid pain increases the likelihood of disability to work and unemployment, decreases wellbeing (9, 10) and doubles health care costs compared with patients with pain without depression (10). This comorbidity is associated with treatment resistance and poor response to treatment when only the depressive symptoms are treated (11–13). Therefore, it is evident that treatment needs to address both depression and pain.

One option for a dual and integrated treatment approach that addresses both depression and pain is collaborative care (CC), which is effective in the treatment of depression (14–17) and pain (18). CC is a framework for multifaceted care, including psychological as well as pharmacological interventions and interdisciplinary collaboration of health professionals, and is applicable in primary, secondary, and tertiary care settings (19). In the Netherlands, the general practitioner (GP) acts as a gatekeeper for patients with mental and physical problems. In the national Depression Initiative, from 2006 to 2012 (20), a CC model was implemented and evaluated in which the GP could collaborate with a nurse care-manager and a consultant psychiatrist for treatment of a depressive disorder in primary care (19). This model has proved to be successful (17, 21–23), and so was a similar model for anxiety disorders (24, 25) and somatoform disorders (26) in the primary care setting. As a result of these positive outcomes, and the cost-effectiveness of the model (27–30), CC including psychiatric consultation was taken into account in a rigorous reorganization cutback of mental health care provisions in the Netherlands (31) and was made the preferred collaboration model for primary care and specialty mental health care in the Netherlands since 2014 by the Ministry of Health and the medical insurance companies. Since then, in order to cope with the increasing demand for mental health care in general practice, a policy change was introduced reimbursing the collaboration of GPs and mental health specialists (e.g., psychiatrists) that is now being implemented widely (32). Patients with mild mental health problems are treated in primary care (with psychopharmacological treatment and/or short-term psychological treatment) and patients with moderately severe mental disorders are treated in basic mental health care, where no more than 12 sessions are offered. Patients with more complex mental health problems are treated in specialized mental health care. In a large randomized controlled trial, antidepressant treatment in combination with a behavioral intervention, such as problem-solving treatment (PST), was more effective in reducing depressive and pain symptoms in primary care patients (33). Antidepressants such as duloxetine are reported to be effective for depressive and pain symptoms (34–39), and appear to be more effective than SSRIs and placebos (36, 40). However, to co-manage pain, the use of analgesics might improve the effect on pain symptoms in patients with depression (33).

**Figure 1:**
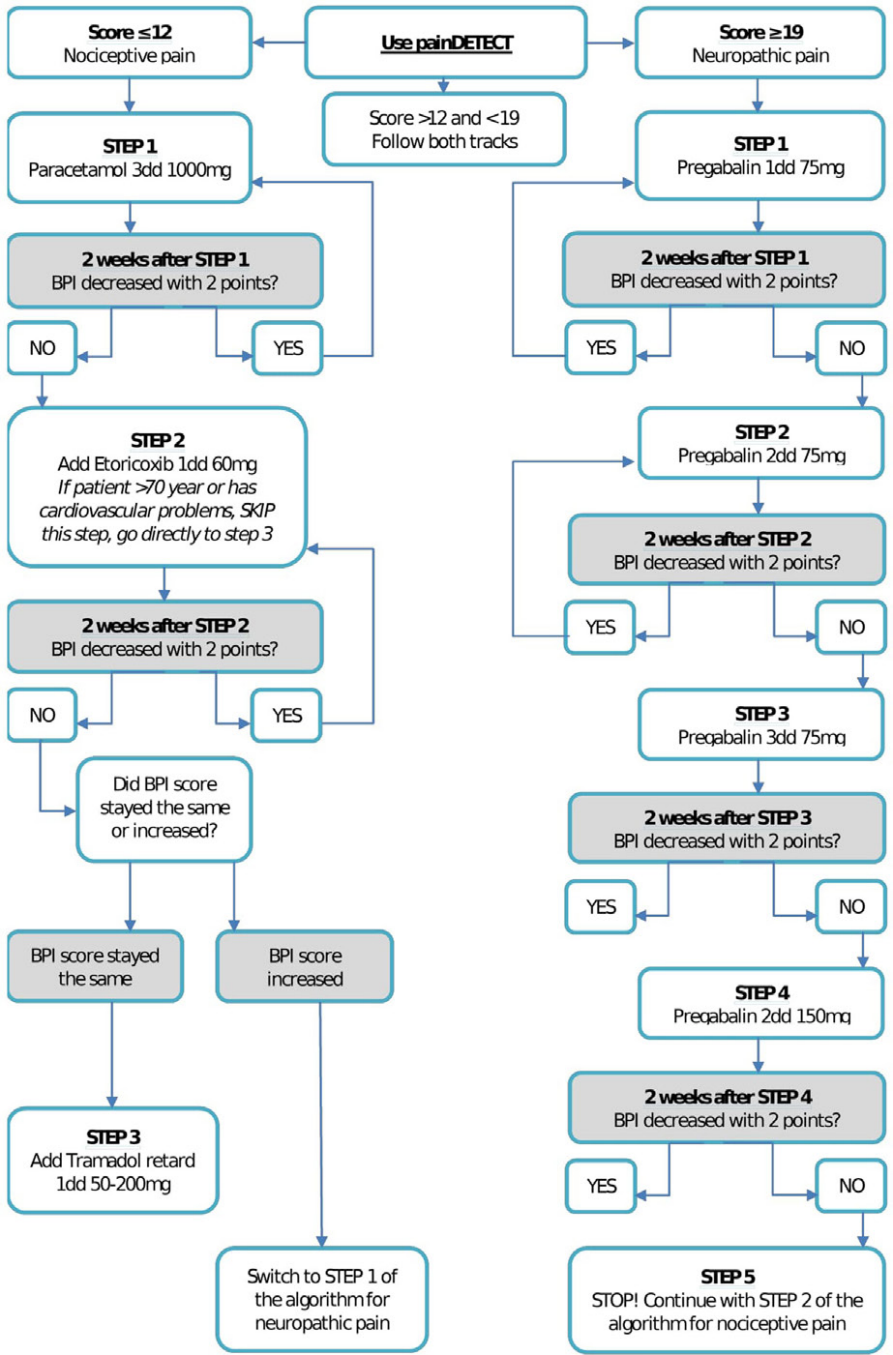
Pain medication algorithm for nociceptive pain (left track), neuropathic pain (right track), and mixed pain (both tracks).

In general, the World Health Organization pain ladder is used in pain treatment, which suggests three steps, the second and third steps of which involve opiates, and antidepressants; pregabalin and mood stabilizers have been suggested as adjuvant medication (41). In the Netherlands, it is growing practice for a psychiatrist to prescribe pain medication. The prescribing practices for opioids, however, particularly as they relate to the management of chronic pain, have been subject to debate (42–47). It is advised to use non-opioid medications in the treatment of pain in patients with depression (44). This new development makes the outcomes of the present study highly relevant, as we developed a new algorithm that might be an attractive alternative to the WHO pain ladder, specifically for the comorbid condition of depressive disorder and pain. This algorithm lays an emphasis on avoiding opiates as much as possible, differentiating between nociceptive pain and neuropathic pain, and prescription of so-called adjuvant medication (i.e., antidepressants and pregabalin) from the start instead of later or only optional in the WHO pain ladder. Furthermore, this new algorithm is embedded in a CC approach involving active monitoring of medication use and its effects. The algorithm is shown in Figure 1.

Pain is common in patients with major depressive disorders (MDDs) and has a negative influence on treatment outcome. Furthermore, pain and depression have a bidirectional association: persons with pain symptoms are at increased risk for depression and those with depressive symptoms report more pain symptoms than persons without depressive symptoms (9). Hence, treatment should address both. Evidence exists for the efficacy of CC against MDD, for the efficacy of consequent use of pain medication against pain, and for the efficacy of duloxetine against both MDD and neuropathic pain. However, their relative effectiveness has not yet been established. Also, in view of miscellaneous results on the effectiveness of duloxetine for pain, a placebo condition in this specific patient group with comorbidity was needed. Hence, the aim of this study was to evaluate the effectiveness of CC with PST, pain medication and duloxetine, and CC with PST, pain medication and placebo, compared with duloxetine alone, on depressive, and pain outcomes. Due to several developments, the enrollment was severely hampered and eventually, the study was prematurely terminated. Surrounding the start of the study, a series of unprecedented changes in the organization and financial reimbursement of mental health care due to cutbacks were introduced in the Netherlands. As a result, fewer patients were treated in specialized mental health care. These developments in mental health care made it difficult for this study to complete enrollment as envisioned. This study is, therefore, of exploratory nature.

## Materials and Methods

### Trial Design

The design of the study has been described in more detail elsewhere (48) but is summarized here. This study was a three-armed, randomized, multicenter, and placebo-controlled trial. The three treatment groups of this study consisted of:

1. CC including pain medication treatment, combined with duloxetine;
2. CC, including pain medication treatment, combined with a placebo;
3. duloxetine only.

The allocation of duloxetine/placebo in the CC groups was double-blind and the duloxetine only group was open label.

### Setting

This study was led by the Clinical Centre for Body, Mind, and Health, and performed within this center and two other mental health institutions in the Netherlands: Arkin, and GGZ inGeest. Inclusion took place between December 2011 and May 2014.

### Ethical Statement

This study was carried out in accordance with the recommendations of the scientific committees of the three participating institutions with written informed consent from all subjects in accordance with the Declaration of Helsinki. All subjects gave written informed consent in accordance with the Declaration of Helsinki. The protocol was approved by the respective scientific committees of the three participating institutions, the medical ethical committee, and by the Central Commission for Human Bound Research (CCMO, dossier number: NL30081.029.10). The trial is listed in the trial registration: http://www.trialregister.nl/trialreg/admin/rctview.asp?TC=1089; NTR number: NTR1089.

### Participants

Consecutive patients presented at three specialized mental health outpatient clinics were screened for MDD and pain; they were asked informed consent and randomly assigned to one of the three treatment groups. Inclusion criteria were a Patient Health Questionnaire depression sub-scale (PHQ9) (49) score of 10 or higher; an MDD classification by Mini Neuropsychiatric Interview (MINI interview); and a brief pain inventory (BPI) (50) score of 3 or higher. Exclusion criteria were: a PHQ9 lower than 10; a BPI score lower than 3; alcohol use of more than 3 units (1 unit = 1 glass of at least 0.25 l) a day; drug abuse or dependence in the last 6 months, defined as current use of any hard drugs (defined by Dutch law, e.g., XTC, cocaine, heroin, magic mushrooms) or cannabis; psychotic symptoms or use of antipsychotic medication that may influence perception of pain; use of St John’s wort (Hypericum Perforatum); pregnancy and breastfeeding; inability to participate in case of too severe language barrier; dementia; history of renal and liver dysfunction for which treatment is needed; uncontrolled hypertension despite treatment for hypertension; suicidal ideation if this constitutes immediate danger and the need for crisis management (48). Patients were asked to stop their current medications, under the supervision of a psychiatrist, before starting with the study.

### Randomization

Each participating patient received a unique identification number. This number was inserted into a specially designed program for randomization. The randomization number (1 for CC + duloxetine, 2 for CC + placebo, and 3 for duloxetine) was digitally sent to the pharmacist of the VU medical center. The pharmacist changed the labels of medication so no information was provided whether the pills were duloxetine or a placebo. The pharmacist could identify the medication by a unique number. After receiving a randomization number, the pharmacist would send four unique medication numbers to the contact of the mental health center where the patient was registered. The contact would ensure the psychiatrist received the correct medication for the included patient. Two people controlled the unique medication numbers assigned to a patient. Study investigators, research coordinators, attending care teams, and the patients were blinded to treatment allocation when randomized into one of the CC groups. Medication in the duloxetine alone group was open label. Randomization in a 1:1:1 fashion was balanced within blocks, in which each treatment arm would occur two times in every six randomizations (48, 51).

### Variables

#### Primary Outcome: Depressive Symptoms

The severity of depressive symptoms was measured with the PHQ9, a brief but validated instrument that scores each of the DSM-IV criteria for MDD (49). Each item is scored from 0 (not at all) to 3 (nearly every day). The total score thus varies from 0 to 27; higher scores indicate higher levels of depressive symptoms. A cut-off score of 10 or higher is recommended to indicate moderate levels of depression (52). Response on depressive symptoms was defined as a 50% reduction of the baseline PHQ9 score at the end of treatment. Remission of depressive symptoms was defined as a score of 5 or lower on the PHQ9 at the end of treatment.

#### Secondary Outcome: Pain Symptoms

To measure pain symptoms, the item for average pain of the BPI (50) was used, measuring average pain severity on a scale of a 10-point scale (0 = no pain; 10 = the most severe pain). The BPI is a self-administered questionnaire that was originally designed to assess cancer pain and shows good validity and reliability (Cronbach alpha.85) (50). The questionnaire is composed of pain drawing diagrams, four items about pain intensity (worst pain, least pain, average pain, and pain right now), two items on pain relief treatment or medication, and one item on pain interference, with seven sub-items (general activity, mood, walking ability, normal walk, relations with other people, sleep, and enjoyment of life). The BPI was completed at every session (seven in total) with the psychiatrist (every other week), who used the BPI score to adjust the medication. Including the score used to screen for eligible patients and the baseline BPI score, a total of nine BPI measures were obtained. Response on pain symptoms was defined as a 50% reduction of the baseline BPI score at the end of treatment. Remission of pain symptoms was defined as a score of 2 or lower on the BPI at the end of treatment.

Classification of nociceptive pain, neuropathic pain, or mixed pain was done using the painDETECT questionnaire that was specifically validated for this purpose (53). The painDETECT classifies subjects into nociceptive, neuropathic, or ambiguous/mixed pain based on a summative score for nine items: neuropathic pain component is unlikely, nociceptive pain is more likely (score ≤ 12), result is ambiguous/mixed (score 13–18), and neuropathic pain component is likely (score ≥ 19). Seven items focus on sensory symptoms for pain [graded from 0 (=never) to 5 (=strongly)], one item focuses on pain course pattern (graded −1 to +1), and one item focuses on pain radiation [graded 0 (for no radiation) or +2 (for radiating pain)]. The total score of the nine-item version ranges from −1 to 38. The painDETECT was originally developed for individuals with low-back pain and showed good sensitivity and specificity (53). The painDETECT was used in the first therapy session in the CC treatment groups and was used to determine the choice of pain medication according to the pain algorithm.

#### Assessments

Before treatment started, all participants completed a baseline self-report questionnaire. During treatment, the patients completed self-report questionnaires BPI, PHQ9, and the Antidepressant Side-Effects Checklist (ASEC-21) (54) provided by the clinician every other week, according to a Case Registration Form (CRF). The ASEC-21 is a checklist to measure the number of side effects due to the use of an antidepressant. The CRF assessments were used for analyses.

#### Medications Used in the Study

In this study, duloxetine was used as an antidepressant. Patients started with 30 mg every day for the first 2 weeks, which was increased to 60 mg for the following 2 weeks. Dependent on the score on the PHQ9, the dose would then be raised to 90 mg (if the PHQ9 score was not decreased with five points) or would remain 60 mg (if the PHQ9 score decreased by at least five points). When after 12 weeks no response or remission was obtained on the PHQ9, a switch to amitriptyline was suggested.

For pain, medication was prescribed according to the nature of pain, for which a distinction was made between nociceptive and neuropathic pain. Figure 1 shows the pain medication algorithm. In short, when a patient reported pain of nociceptive nature, paracetamol 1,000 mg 3dd was prescribed as a first step. When pain symptoms did not decrease in the following 2 weeks, etoricoxib 60 mg 1dd was added. Tramadol 50–200 mg 1dd was added as a third step if the severity of pain symptoms stayed the same after the use of etoricoxib. If the severity increased, a switch to the algorithm for neuropathic pain was made. For neuropathic pain, pregabaline was prescribed, starting with 75 mg 1dd and an increase of 75 mg 1dd every 2 weeks if the severity of pain symptoms did not decrease.

### Intervention

A comprehensive description of the intervention can be found elsewhere (48). Here, we will provide a summary of the intervention.

In all three treatment groups, the intervention lasted 12 weeks. All participants completed the painDETECT questionnaire (to assess nociceptive and neuropathic pain), the PHQ9, and the BPI at baseline. During treatment, assessments of PHQ9 and BPI were performed every other week. Patients who did not show up for three or more sessions were registered as non-compliant.

Every week, each patient had a session with a psychologist, and every other week a session with a psychiatrist. In case of treatment response (50% reduction of the initial score), but non-remission, as indicated by a score of >5 on the PHQ9 after 12 weeks of treatment, the patient was referred to the GP with subsequent antidepressant treatment and pain medication advice. If after 12 weeks (after de-blinding the medication code) no treatment response had occurred on severity of depressive symptoms, as indicated as less improvement than 50% on the initial PHQ9 score, the patient was not referred to the GP but referred instead for further specialized mental health care. During the treatment, the patient worked through a self-help manual containing information about depression and pain symptoms, antidepressant medication and relaxation techniques, and was guided by the psychologist. The psychologist treated patients using PST, a brief, structured psychological intervention, consisting of seven stages (55). PST is based on the fact that emotional symptoms are often associated with problems in daily life, and it encourages patients to formulate practical ways of dealing with such problems (55). The goal of PST is to teach patients to use their own skills and resources to function better and, thus, improving their coping skills. The psychiatrist prescribed and monitored pain medication and duloxetine, and monitored possible side effects of the medication (48). In all treatment groups, a CRF was used to monitor treatment and guide the therapist to carry out treatment per protocol. The psychologist and psychiatrist both had a CRF made for a specific patient and for their specific treatment. Both the CRF of the psychiatrist and of the psychologist were divided into sections, with each section representing a session. Each session started with the steps that had to be followed for that specific session. The CRF for the psychiatrist was as follows: each session started by asking about the presence and severity of 21 common antidepressant side effects using the ASEC-21. When an adverse event not mentioned on the ASEC-21 was attributed to the antidepressant therapy, the therapist could write this down. Subsequently, the depressive and pain symptoms were monitored using the PHQ9 and the BPI. The scores on these questionnaires were used to determine the next step in the medication algorithms, which could also be found in the current section of the CRF. The algorithm for pain medication is shown in Figure 1. The CRF for the psychologist was as follows: each session consisted of PST, and every session one problem was addressed that bothered the patient the most. These sessions took place every week.

#### Collaborative Care

In the CC groups, duloxetine or placebo was prescribed, and pain medication was administered according to an algorithm specifically designed for this study. This algorithm avoids opiate prescription as much as possible, which is considered as an alternative to the current WHO pain ladder, with paracetamol, COX inhibitors, and pregabalin as steps before opiates are started.

In the CC groups, duloxetine (or placebo) and pain medication were administered by a psychiatrist, using an algorithm that is shown in Figure 1.

Collaborative care was simultaneously provided by a team of a care-manager (psychologist) and a psychiatrist. Only patients in the CC groups were treated by all specialists. The care-manager was responsible for PST, a brief, structured psychological intervention.

#### Duloxetine Only

The patients in the duloxetine only group received treatment of the psychiatrist who used a CRF and the duloxetine algorithm in prescribing duloxetine.

#### Compliance

Patients had to show up at least 80% of the sessions to be considered as compliant to treatment. Patients who did not show up for three or more sessions (20% of the sessions) were registered as non-compliant (48).

### Statistical Methods

Due to the premature termination of this study, not all data could be used for analyses, and the follow-up period of 12 weeks was used instead of the planned 12 months. However, based on several studies that were performed after the initial planning of our study, in which we assumed 189 patients would be needed to enable us to find a result, we recalculated the power (56) of the study to estimate if analysis of the CRF data might be useful. We found three relevant studies. A study reporting treatment response on the PHQ9 comparing duloxetine with placebo (57) found 66% response in duloxetine versus 25% in placebo; to find a similar effect, assuming alpha 0.05 and power 80%, the *n* should be 24 patients for each treatment arm. Two other trials, one comparing duloxetine to placebo (35), and an RCT comparing CC versus care as usual (17) found similar differences in effect. Hence, as we had 20 patients per arm, we decided to evaluate a preliminary estimate of efficacy of CC and duloxetine with pain medication as intended previously, with the data from the CRF assessments, as it might be possible to find an effect. Nevertheless, these should be considered exploratory analyses rather than hypothesis testing. A full report on the factors that hampered this study and led to the premature termination of this study can be requested from the corresponding author. The 12 weeks CRF assessments were available only for limited analyses. Although the sample size was smaller than intended, explorative, intention-to-treat and per protocol, multilevel regression analyses (MLAs) were performed.

Descriptive analyses were performed to describe the sample at baseline, regarding gender, age, compliance, severity of depressive symptoms, and pain severity. Differences between treatment groups and between compliant and non-compliant patients were analyzed regarding baseline characteristics. All linear variables were normally distributed (normality was tested with the Shapiro–Wilk test). Although the required sample sizes were not reached, we performed explorative, intention-to-treat, MLAs. We evaluated the effect of time for the whole sample, the effect of the treatment group, and the effect of the treatment group over time in terms of depressive symptoms and in terms of pain symptoms over a 16-week period (from moment of screening to the end of the therapy sessions). Quadratic functions of time were included to examine whether there was an initial increase in outcomes followed by a decrease (negative quadratic function), or the other way around (positive quadratic function). Third, explorative, per-protocol MLAs were used to evaluate the effect in case of compliance. Data from all 60 participants were used in the analyses. In the per-protocol analyses, the compliant and non-compliant patients were separately analyzed to examine differences between these groups. There were no dropouts. The duloxetine only group was used as the reference group in all analyses because we expected the CC groups to be the most effective compared with the duloxetine only group. The number of side effects that were reported on the ASEC-21 is presented for the total sample and for all the treatment groups. Between group analyses (one-way ANOVA, with LSD *post hoc* test) were performed to examine whether the number of side effects reported would differ between the treatment groups.

## Results

### Participant Flow

**Figure 2:**
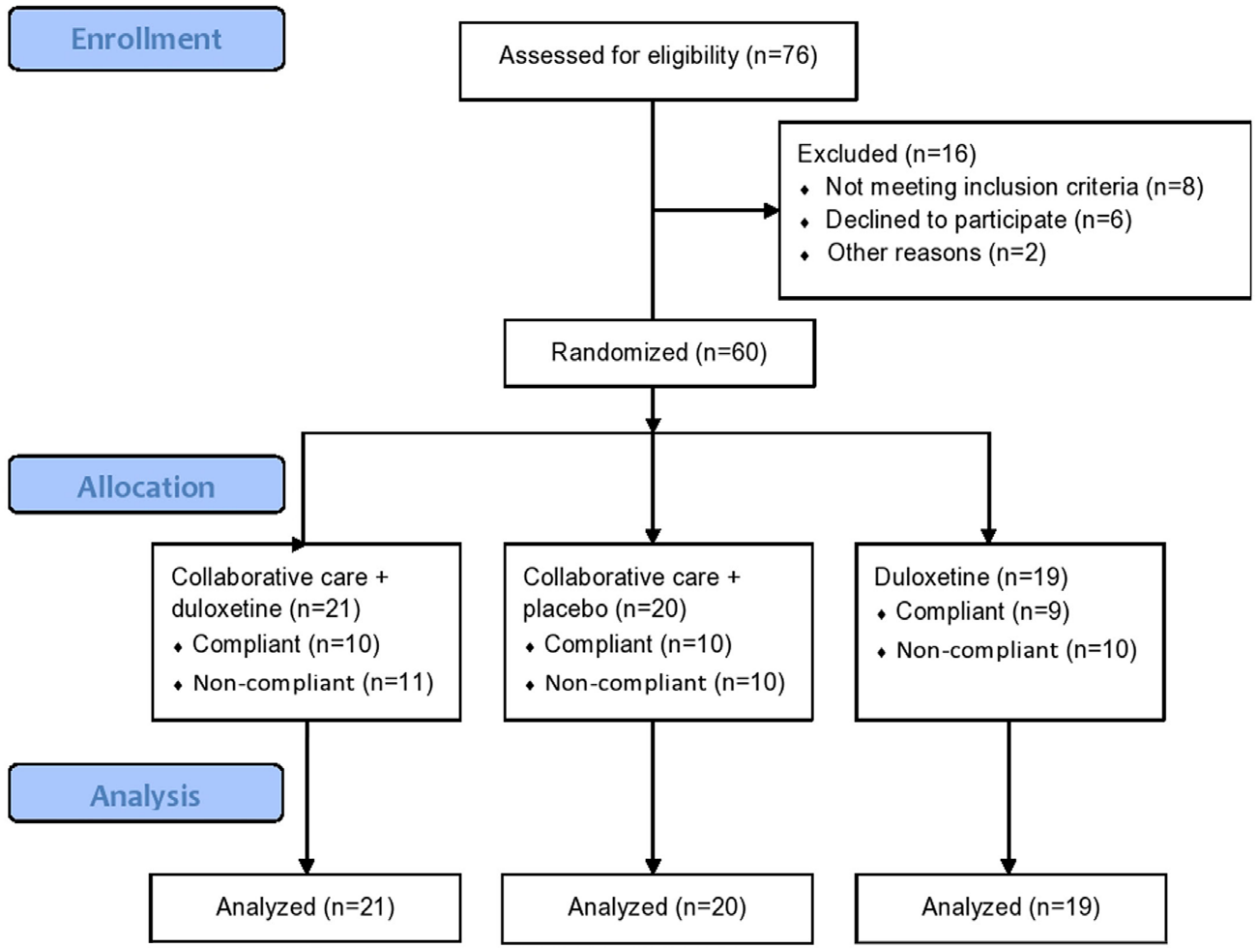
Overview of inclusion, divided in three research groups and number of non-compliant (having missed three or more sessions) and compliant patients.

The inclusion and follow-up of patients is shown in the flowchart in Figure 2. Of the 76 eligible patients, 16 were excluded for several reasons, including having an elevated risk of suicide or not being fluent in Dutch. Twenty-one patients (35%) were randomly assigned to the CC with duloxetine condition, 20 patients (33.3%) to the CC with placebo condition, and 19 patients (31.7%) to the duloxetine alone condition. Of the total sample, 29 patients (48.3%) were compliant, whereas 31 patients (51.7%) were non-compliant for several reasons: (a) side effects; (b) did not want the medication; (c) did not want to continue in the study; (d) moved to another city; and (e) needed/wanted other care. Of all included patients, follow-up measurements were attained during the intervention period, hence no loss of follow-up occurred in this time frame.

### Baseline Characteristics

Table 1 shows the baseline characteristics of the total sample and the three treatment groups. The total sample consisted of more women than men, with a mean age of 43. Mean score of the PHQ9 was 17.45 and of the BPI 6.92. Most patients reported neuropathic pain (*n* = 20), and only five patients reported pain of both a nociceptive and neuropathic nature. No significant differences were found between the three treatment groups regarding gender, age, depressive symptoms, and pain symptoms. The two CC groups did not differ regarding the nature of pain. Table 2 shows the baseline characteristics of compliant and non-compliant patients. Both groups consisted of more women than men. Mean age was 41.9 and 44.4 for compliant and non-compliant, respectively. Mean PHQ9 score was somewhat higher for non-compliant patients (mean = 17.9) than for compliant patients (mean = 17.0), and mean score of the BPI was higher for compliant patients (mean = 7.2) than for non-compliant patients (mean = 6.7). In both groups, an equal number of patients reported pain of nociceptive nature (*n* = 7) and of neuropathic nature (*n* = 10). No significant differences were found between compliant versus non-compliant patients regarding gender, age, treatment condition, severity of depressive and pain symptoms, and nature of pain.

**Table 1:** Gender, age and mean of pain, and depressive symptoms at baseline of the total sample and three treatment conditions.

	Total sample, *N* = 60	CC + duloxetine, *n* = 21 (35%)	CC + placebo, *n* = 20 (33.3%)	Duloxetine, *n* = 19 (31.7%)
**Sample characteristics**				
Female gender, *n* (%)	36 (60%)	15 (71.4)	10 (50)	11 (57.9)
Age, mean (SD)	43.2 (12.4)	41.9 (12.2)	41 (12.9)	46.9 (11.7)
PHQ9, mean (SD)	17.5 (4.3)	17.2 (4.2)	17.5 (5.0)	17.7 (3.7)
BPI, mean (SD)	6.9 (1.7)	6.7 (2.1)	7.1 (1.4)	7.0 (1.5)
**Nature of pain, *n*(%)^a^**				
Nociceptive Neuropathic Mixed	14 (23.3%) 20 (33.3%) 5 (8.3%)	7 (33.3%) 10 (47.6%) 3 (14.3%)	7 (35%) 10 (50%) 2 (10%)	

*^a^Patients in the duloxetine alone group (*n* = 19) did not receive pain medication, therefore, no information was obtained on nature of pain for those patients. Of two other patients, information regarding nature of pain was missing*.

**Table 2:** Gender, age, treatment condition, depressive and pain symptoms, and nature of pain at baseline of compliant (*n* = 29) and non-compliant (*n* = 31) patients.

	Compliant patients (*n* = 29)	Non-compliant patients (*n* = 31)
**Sample characteristics**		
Female gender, *n* (%)	16 (55.2)	20 (64.5)
Age, mean (SD)	41.9 (11.4)	44.4 (13.4)
**Treatment condition, *n* (%)**		
CC + duloxetine CC + placebo Duloxetine	10 (34.5%) 10 (34.5%) 9 (31.0%)	11 (35.5%) 10 (32.3%) 10 (32.3%)
PHQ9, mean (SD)	17.0 (4.0)	17.9 (4.6)
BPI, mean (SD)	7.2 (1.6)	6.7 (1.7)
**Nature of pain, *n*(%)^a^**		
Nociceptive Neuropathic Mixed	7 (24.1%) 10 (34.5%) 3 (10.3%)	7 (22.6%) 10 (32.3%) 2 (6.5%)

*^a^Patients in the duloxetine alone group (*n* = 19) did not receive pain medication, therefore, no information was obtained on nature of pain for those patients. Of two other patients, information regarding nature of pain was missing*.

### Intention-to-Treat Analysis

**Figure 3:**
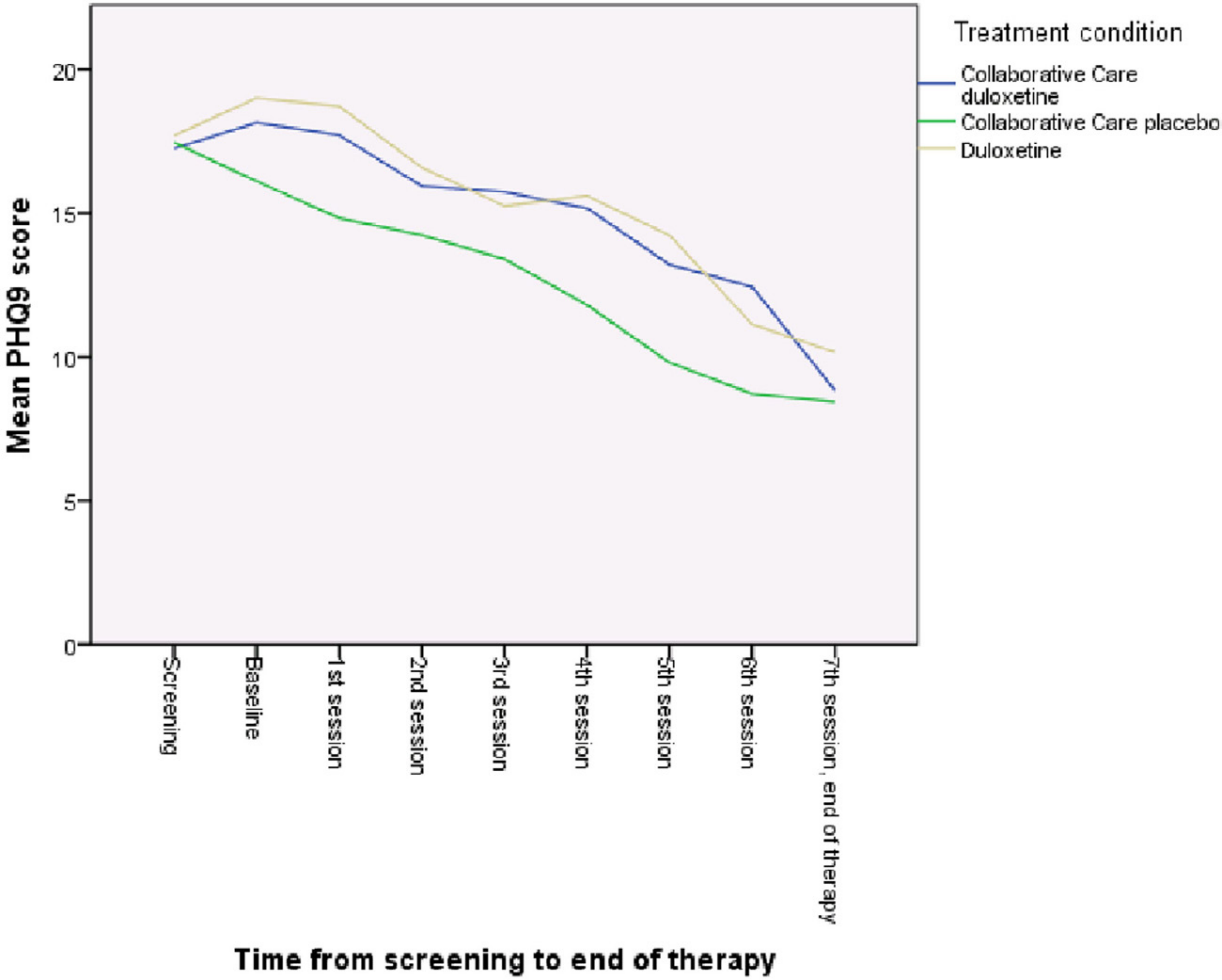
Mean Patient Health Questionnaire 9 (PHQ9) score of the three treatment groups over time.

**Figure 4:**
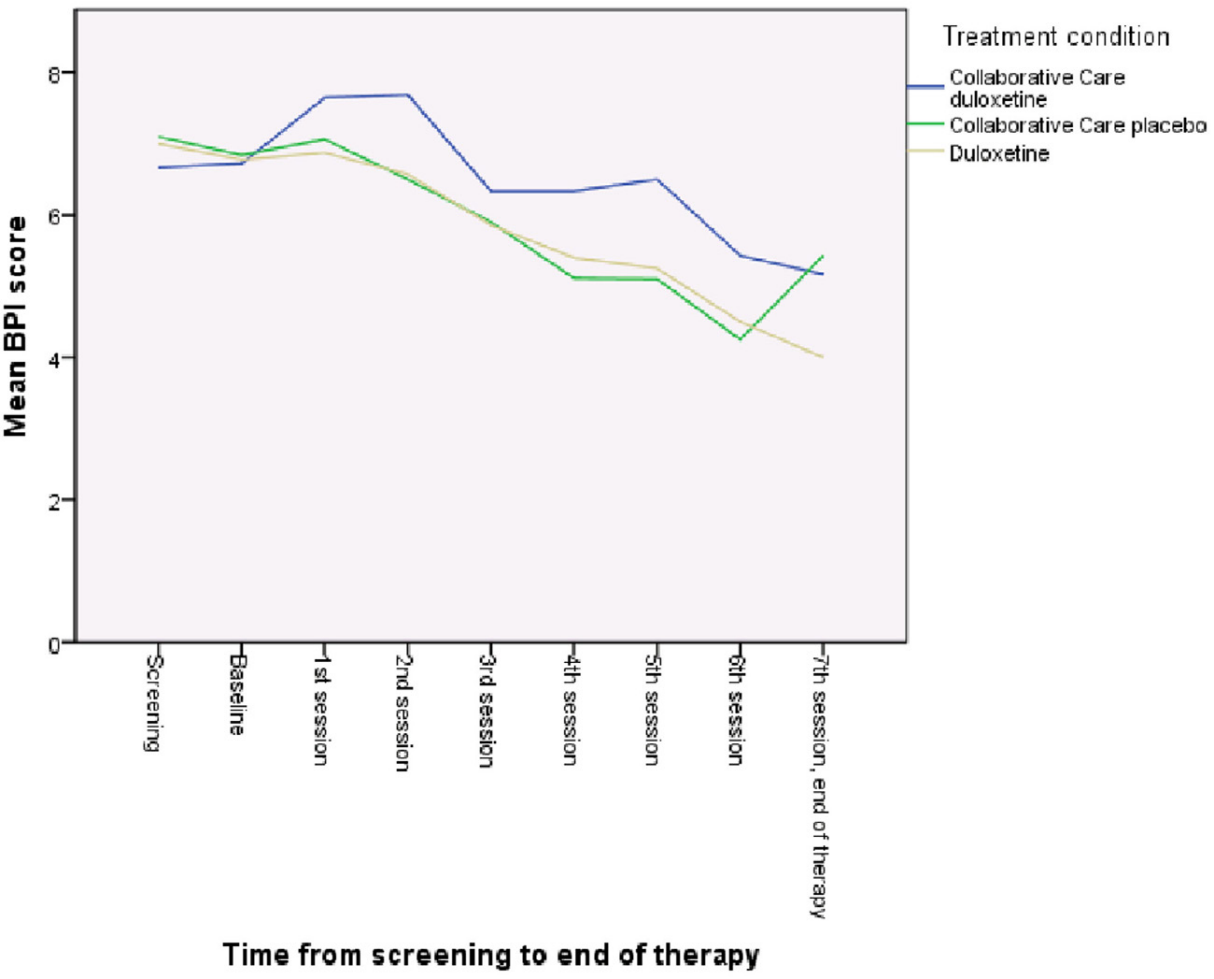
Mean brief pain inventory (BPI) score of the three treatment groups over time.

As is shown in Table 3, patients in all treatment groups reported significant lower depressive (*b* = −0.34; *p* = < 0.001) and pain (*b* = −0.07; *p* = 0.01) symptoms after therapy (Table 3). ITT analysis showed that, for pain, both CC treatment groups did not show significantly better results than the duloxetine alone group. For the outcome of depression, the CC with placebo condition showed the fastest decrease compared with the duloxetine alone group (*b* = −0.78; *p* = 0.01), but this effect diminished at the end of treatment (Figure 3). Figures 3 and 4 show the mean PHQ9 and mean BPI scores of the three treatment groups over time, respectively.

**Table 3:** Intention-to-treat, multilevel analyses comparing the CC + duloxetine and CC + placebo treatment groups with the duloxetine alone treatment group for depressive and pain symptoms.

	Total sample, *N* = 60	CC + duloxetine, *n* = 21	CC + placebo, *n* = 20	Duloxetine, *n* = 19
		
	β (95% CI)	β (95% CI)	β (95% CI)	
**Depression**				
Effect of time	**−0.34 (−0.47 to −0.20)^a^**			Reference
Effect of treatment group		−0.31 (−3.16 to 2.54)	−0.54 (−3.43 to 2.34)	Reference
Effect of treatment group and time		−0.28 (−0.83 to 0.27)	**−0.78 (−1.33 to −0.22)^a^**	Reference
Effect of treatment group and time (quadratic)		0.02 (−0.01 to 0.05)	**0.04 (0.01 to 0.07)^a^*p* = 0.01**	Reference
**Pain**				
Effect of time	**−0.07 (−0.13 to −0.02)^a^**			Reference
Effect of treatment group		−0.27 (−1.24 to 0.71)	0.26 (−0.73 to 1.24)	Reference
Effect of treatment group and time		0.15 (−0.09 to 0.40)	−0.13 (−0.39 to 0.12)	Reference
Effect of treatment group and time (quadratic)		−0.01 (−0.02 to 0.01)	0.01 (−0.01 to 0.02)	Reference

*^a^Significant at the 0.05 level; CC, collaborative care; time, time from screening to end of therapy sessions; significant results are bold*.

### Per-Protocol Analysis

Per-protocol analysis with the compliant patients showed comparable results as the ITT analysis (Table 4). Non-compliant patients did not improve over the 12-week period for depressive or pain symptoms (results not shown).

**Table 4:** Explorative, per protocol, multilevel analyses comparing the CC + duloxetine, and CC + placebo treatment groups with the duloxetine alone treatment group for depressive and pain symptoms for patients who were compliant.

	Total sample, *N* = 60	CC + duloxetine, *n* = 21	CC + placebo, *n* = 20	Duloxetine, *n* = 19
		
	β (95% CI)	β (95% CI)	β (95% CI)	
**Depression**				
Effect of time	**−0.37 (−0.53 to −0.22)^a^**			Reference
Effect of treatment group		−0.52 (−4.58 to 3.54)	0.19 (−3.83 to 4.22)	Reference
Effect of treatment group and time		−0.17 (−0.86 to 0.52)	**−0.72 (−1.40 to −0.04)^a^**	Reference
Effect of treatment group and time (quadratic)		0.02 (−0.02 to 0.05)	**0.04 (0.01 to 0.07)^a^**	Reference
**Pain**				
Effect of time	**−0.13 (−0.20 to −0.06)^a^**			Reference
Effect of treatment group		−0.77 (−2.17 to 0.64)	0.11 (−1.27 to 1.49)	Reference
Effect of treatment group and time		0.20 (−0.14 to 0.54)	−0.15 (*−*0.48 to 0.18)	Reference
Effect of treatment group and time (quadratic)		−0.01 (−0.02 to 0.01)	0.01 (−0.01 to 0.03)	Reference

*^a^Significant at the 0.05 level; CC, collaborative care; time, time from screening to end of therapy sessions; significant results are bold*.

### Adverse Effects

Table 5 shows the number of patients who reported adverse effects, measured with the ASEC-21. Most patients experienced drowsiness, a dry mouth, nausea, feeling light-headed, insomnia, headache, and sweating. In the placebo condition, adverse effects were also experienced. In this treatment condition, most patients experienced insomnia, drowsiness, feeling light-headed, a dry mouth, and sweating. In the duloxetine alone condition, headache was the most reported adverse effect. Adverse effects reported by patients in the CC with duloxetine treatment group are due to both duloxetine and the pain medication. Adverse effects reported by patients in the CC with placebo treatment group are most likely due to only the pain medication. In the duloxetine alone treatment group, the reported adverse effects are due to duloxetine. Patients in the CC + duloxetine treatment group reported significantly more side effects than patients in the other treatment groups (*F* = 6.27; *p* = < 0.01). No significant differences were found between the duloxetine alone treatment group and the CC + placebo treatment group.

**Table 5:** Number of patients who reported side effects of duloxetine/placebo measured with the ASEC-21.

Symptom	Total sample (*N* = 60), *n*(%)	CC + duloxetine (*n* = 21), *n*(%)	CC + placebo (*n* = 20), *n*(%)	Duloxetine (*n* = 19), *n*(%)
Drowsiness	28 (47)	11 (52)	8 (40)	9 (47)
Dry mouth	27 (45)	12 (57)	7 (35)	8 (42)
Nausea or vomiting	27 (45)	13 (62)	5 (25)	9 (47)
Feeling light-headed on standing	27 (45)	13 (62)	8 (40)	6 (32)
Insomnia	26 (43)	11 (52)	9 (45)	6 (32)
Headache	25 (42)	9 (43)	6 (30)	10 (53)
Sweating	23 (38)	7 (33)	7 (35)	9 (47)
Yawning	18 (30)	8 (38)	3 (15)	7 (37)
Decreased appetite	17 (28)	7 (33)	5 (25)	5 (26)
Tremor	15 (25)	7 (33)	5 (25)	3 (16)
Blurred vision	14 (23)	7 (33)	4 (20)	3 (16)
Feeling like the room is spinning	13 (22)	8 (38)	4 (20)	1 (5)
Weight gain	13 (22)	7 (33)	3 (15)	3 (16)
Constipation	12 (20)	5 (24)	2 (10)	5 (26)
Problems with sexual function	10 (17)	6 (29)	0 (0)	4 (21)
Diarrhea	9 (15)	3 (14)	5 (25)	1 (5)
Increased appetite	9 (15)	5 (24)	1 (5)	3 (16)
Palpitations	9 (15)	6 (29)	1 (5)	2 (11)
Disorientation	8 (13)	5 (24)	1 (5)	2 (11)
Problems with urination	7 (12)	3 (14)	1 (5)	3 (16)
Increased body temperature	3 (5)	0 (0)	3 (15)	0 (0)
Average number of side effects	16	7.3^a^	4.2	4.7

*Adverse effects reported by patients in the collaborative care with duloxetine treatment group are due to both duloxetine and the pain medication. Adverse effects reported by patients in the collaborative care with placebo treatment group are most likely due to only the pain medication. In the duloxetine alone treatment group, the reported adverse effects are due to duloxetine*.

*^a^Significantly different at 0.05 level, using one-way ANOVA with LSD post hoc test, compared with CC + placebo and compared with duloxetine only*.

## Discussion

We carried out a randomized controlled trial, testing and comparing the effectiveness of three active treatments among patients with moderately severe MDD and comorbid pain. This is a notoriously hard group of patients to engage and treat, and the prognosis of both dimensions of symptoms, when left untreated, is unlikely to be favorable (11–13). Our first result is that, at the end of the treatment, patients in all treatment groups had significantly less pain and depressive symptoms. Considering pain, there were no significant differences between the three treatments. However, the depressive symptoms decreased faster among patients in the CC with placebo group than among patients in the duloxetine only group. For patients who were non-compliant, depressive, and pain symptoms did not decrease, which indicates that the treatments provided did play a role in the outcomes. Hence, our findings suggest that in comorbid MDD and pain, compliance of the patient and placebo effects are more crucial than choice of one of the three treatments explored in this RCT.

A review of 79 studies comparing CC with routine care or alternate treatments found that CC leads to greater improvement than care as usual in depression outcomes in the short term as well as the long term (14). Our results also show a significant improvement of depressive symptoms when CC is offered. The effect occurs faster in the CC + placebo arm. However, at the end of the intervention period, the outcome is similar in the three treatment arms. In our study, duloxetine had no surplus value for patients in the CC groups, both for depressive symptoms and for pain, which was against our expectations based on studies examining the effect of duloxetine on depression and pain (34, 36, 40). Our findings do correspond with a meta-analysis of duloxetine’s purported analgesic effects on depressed patients, in which the analgesic effects of duloxetine were not supported (58).

The finding that the CC with placebo condition seems to be the most optimal condition in this study is intriguing. A possible explanation might be the placebo effect, which could increase the effect of CC, at least at the beginning of therapy. Patients in both CC groups most likely have an expectation of a reward (i.e., expectation of less pain and depressive symptoms) and had experience with the use of similar medications, have learned the (positive) effects of these medications, and are therefore more likely to experience a positive effect (classical conditioning), which might contribute to less psychological and physical symptoms (59, 60). Considering that side effects of an antidepressant tend to occur mostly in the first few weeks of use might be a possible explanation as experiencing side effects can increase the likelihood of a poorer treatment outcome (61). Hence, we examined the occurrence of side effects in the three treatment groups. As described in the results, no significant differences in terms of side effects were found between the duloxetine alone and the CC + placebo treatment groups, but treatment response was fastest in the CC + placebo treatment group. Patients in the CC + duloxetine treatment group, however, reported significantly more side effects than patients in the other two treatment groups. This suggests that the fast treatment response in the CC + placebo treatment group has to do with receiving a high level of care and attention (CC) and with experiencing less side effects (placebo). We would like to encourage similar studies among larger sample sizes and longer follow-up periods to explore the possible mechanism further and to enhance the generalizability of our results.

This study is a first step to establish an effective treatment for the combination of depression and pain. A major strength of this study is the use of an algorithm for pain medication, which distinguishes nociceptive pain from neuropathic pain. A WHO pain ladder algorithm does exist, but that algorithm focuses mainly on nociceptive pain. An algorithm for neuropathic pain was not yet available. The results indicate that active pain management in CC with COX inhibitors and pregabalin as alternatives to tramadol or other opiates might provide an attractive alternative to the current WHO pain ladder as it avoids opiate prescription as much as possible. Recently, it was suggested that adaptations of the original WHO pain ladder are needed (62). For example, opioids should be considered as adjuvant medications instead of the principal medication for the treatment of pain (62), and in other research, it is discussed to select medication for patients with neuropathic pain with good therapeutic effects and a small likelihood of side effects, such as pregabalin (63). Our algorithm is, therefore, a next step in the adaptation of the existing pain ladder and might be useful for health professionals around the world who treat patients with pain symptoms. Another strength is the use of a placebo condition in combination with a three-armed design that enabled us to explore the relative effect of CC, pain medication algorithm, and duloxetine alone.

This study has several limitations that need to be addressed, however. All treatment groups had some form of active treatment, which makes spontaneous recovery or the natural fluctuation of symptoms hard to address. However, in a large observational study, severity of pain did not change over time, and even increased in those persons with depressive symptoms when compared with healthy persons (64), so the decrease in depressive and pain symptoms as found in this study might be accounted for by the treatment offered. Also, the fact that patients in our study who did not comply with the treatment did not improve is an indication that improvement of depressive and pain symptoms was associated with treatment. In this study, we examined the effectiveness of CC, in which we included a new pain management program. We could, however, not assess the impact of this pain management program on treatment effect separately. Therefore, a need exists for future studies also including a treatment group consisting of CC including duloxetine, but no pain management program, to examine whether a pain management program increases treatment effect on top of CC and duloxetine. This also applies to the effect of CC alone. To measure the effectiveness of CC, future research needs to include a treatment group consisting of only CC. As mentioned before, the sample size of this study is small due to the reasons described. Although significant results were found, these need to be interpreted with some caution. Research with a larger sample size is needed for generalizability of the results. This study used a follow-up period of 12 weeks, so no inferences could be made regarding the long-term effect of these treatments after end of treatment. However, in a review comparing CC with other treatments, CC had a significant effect on depression outcomes for up to two years after treatment (14), and it is, therefore, plausible that the therapeutic effects found in this study might also last for a longer period than used in this study. To study long-term effects and the cost-effectiveness of these treatments, which was envisioned in the original design, both a larger sample size and longer follow-up period are needed. Moreover, to effectively conduct such a study, if possible, future health policies of government and health insurance companies should be taken into account, as these can influence the feasibility of the study greatly. Including more mental health organizations might also decrease the inclusion period and increase the number of inclusions, which might add to a successful completion of the study. Starting with a feasibility study can help in successfully conducting a full-scale randomized controlled trial for CC for pain and depression in the Netherlands.

The findings from this study are better thought of as hypothesis forming rather than hypothesis testing, and it would be necessary to see the conclusions replicated in future trials. However, CC with active pain management seems promising in the treatment of depression when pain is present and our pain medication algorithm might provide an attractive alternative to the current WHO pain ladder. This needs further exploration in full-scale trials.

## Ethics Statement

This study was carried out in accordance with the recommendations of the scientific committees of the three participating institutions with written informed consent from all subjects in accordance with the Declaration of Helsinki. All subjects gave written informed consent in accordance with the Declaration of Helsinki. The protocol was approved by the respective scientific committees of the three participating institutions, the medical ethical committee, and by the Central Commission for Human Bound Research (CCMO, dossier number: NL30081.029.10). The trial is listed in the trial registration (http://www.trialregister.nl/trialreg/admin/rctview.asp?TC=1089; NTR number: NTR1089).

## Author Contributions

EH, CF-C, and JD conceived the initial idea for the present study and all authors contributed to its planning, including defining the aims, variables of interest, and analysis strategy. Analyses were done by EH and LT, but all authors had access to the statistical outputs. EH drafted the article and all authors contributed to revisions. All authors approved the final manuscript. All authors agreed to be accountable for all aspects of the work in ensuring that questions related to the accuracy or integrity of any part of the work are appropriately investigated and resolved.

## Conflict of Interest Statement

CF-C and JD received financial support and the study medication from Eli Lilly; AB reports personal fees from Lundbeck, outside the submitted work; all other authors have nothing to disclose.
